# Treatment of *MDR1* Mutant Dogs with Macrocyclic Lactones

**DOI:** 10.2174/138920112800399301

**Published:** 2012-05

**Authors:** Joachim Geyer, Christina Janko

**Affiliations:** Institute of Pharmacology and Toxicology, Faculty of Veterinary Medicine, Justus Liebig University of Giessen, Frank-furter Str. 107, 35392 Giessen

**Keywords:** Dog, ivermectin, ivermectin-sensitive Collie, MDR1, milbemycin oxime, moxidectin, P-glycoprotein, pharmacogenetics.

## Abstract

P-glycoprotein, encoded by the *multidrug resistance* gene *MDR1*, is an ATP-driven drug efflux pump which is highly expressed at the blood-brain barrier of vertebrates. Drug efflux of macrocyclic lactones by P-glycoprotein is highly relevant for the therapeutic safety of macrocyclic lactones, as thereby GABA-gated chloride channels, which are confined to the central nervous system in vertebrates, are protected from high drug concentrations that otherwise would induce neurological toxicity. A 4-bp deletion mutation exists in the *MDR1* gene of many dog breeds such as the Collie and the Australian Shepherd, which results in the expression of a non-functional P-glycoprotein and is associated with multiple drug sensitivity. Accordingly, dogs with homozygous *MDR1* mutation are in general prone to neurotoxicity by macrocyclic lactones due to their increased brain penetration. Nevertheless, treatment of these dogs with macrocyclic lactones does not inevitably result in neurological symptoms, since, the safety of treatment highly depends on the treatment indication, dosage, route of application, and the individual compound used as outlined in this review. Whereas all available macrocyclic lactones can safely be administered to *MDR1* mutant dogs at doses usually used for heartworm prevention, these dogs will experience neurological toxicity following a high dose regimen which is common for mange treatment in dogs. Here, we review and discuss the neurotoxicological potential of different macrocyclic lactones as well as their treatment options in *MDR1 *mutant dogs.

## P-GLYCOPROTEIN: A MULTIDRUG EFFLUX TRANSPORTER

The multidrug carrier P-glycoprotein (P-gp), encoded by the *MDR1* (*ABCB1*) gene, belongs to the family of membrane bound ATP-binding cassette (ABC) transporters [1]. P-glycoprotein is an ATP-driven efflux pump that confers *multidrug resistance* (MDR) to cancer cells by actively extruding a wide range of structurally unrelated chemotherapeutic compounds from the cell. Juliano &amp; Ling [2] first isolated P-gp as a membrane glycoprotein of approximately 170-kDa from chemotherapeutic drug-resistant Chinese hamster ovary cells that were selected for colchicine resistance and identified this protein as a major part of the functional multidrug resistance of these cells by limiting their permeability into the cell (P-gp, permeability glycoprotein). Many years later a cDNA was isolated from a multidrug-resistant carcinoma cell line, selected for its resistance to colchicine, vinblastine and doxorubicin, and was shown to encode P-gp [3,4]. Subsequently, the name *MDR1* was established for the gene as well as for the encoded P-gp. Later on, by using bioinformatic approaches, the *MDR1* gene was phylogenetically classified as member B1 of the ABC transporter superfamily [5]. The *MDR1* (*ABCB1*) gene exist in all mammals analysed to date including the dog, with the peculiarity that this gene is duplicated in rodent genomes (referred to as *mdr1a* and *mdr1b*).

Many years of research on P-gp focused on the chemotherapeutic resistance of tumour cells and so the first P-gp substrates identified were cytostatic drugs [6,7]. Today, it is known that P-gp has a broader substrate specificity and transports a large number of structurally unrelated drugs and xenobiotics including anticancer drugs (*e.g.*, vinca alkaloids, paclitaxel, doxorubicin), immunosuppressants (cyclosporine, tacrolimus), antiparasitic agents (ivermectin, moxidectin, selamectin, milbemycin oxime), antimicrobial agents (*e.g.*, erythromycin, rifampicin, ketoconazole, levofloxacin), cardiac drugs (*e.g.*, digoxin, verapamil, diltiazem, quinidine, talinolol, losartan), opioids (*e.g.*, morphine, loperamide, butorphanol, fentanyl), steroid hormones (cortisol, dexamethasone, aldosterone) and many others (*e.g.*, cimetidine, fexofenadine, acepromazine, domperidone, ondansetron) [8-11]. Most P-gp substrates are hydrophobic molecules and partition into the plasma membrane from where they are effluxed by P-gp. Accordingly, P-gp has been thought of as 'hydrophobic vacuum cleaner' for hydrophobic molecules which are embedded into the plasma membrane [12]. This type of substrate recognition makes P-gp a highly effective efflux pump, preventing the cellular entry of toxic compounds [13].

Apart from neoplastic tissues, P-gp shows high expression in the apical (luminal) membranes of epithelial cells lining the lower gastrointestinal tract, in the brush border of renal proximal tubules, in the canalicular membrane of hepatocytes and in capillary endothelial cells in the brain and testes. Furthermore, P-gp expression was found in the placenta, the adrenal cortex and CD34+ hematopoietic stem cells [14-20]. According to this expression pattern, it has been shown that P-gp limits drug absorption in the gastrointestinal tract and promotes drug elimination in the liver, kidney and intestine. Furthermore, P-gp restricts drug uptake into cells and tissues, in particular their permeation across the blood-brain barrier Fig. (**1**). Taken altogether, P-gp has an important protective function for the organism by eliminating potentially toxic compounds from the body and preventing their entry into the brain and organs of reproduction [21-23].

The important role of P-gp in protecting the brain from the penetration of drugs across the blood-brain barrier is highly relevant for the treatment of mammals with macrocyclic lactones. In parasitic lower organisms, macrocyclic lactones bind with high affinity to glutamate-gated and GABA-gated chloride ion channels which are widespread in the nervous system of arthropods and nematodes, resulting in an inhibition of nerve activity, flaccid paralysis and death [24,25]. However, the situation is completely different in mammals where neuronal glutamate-gated chloride channels are absent and GABA-gated chloride channels are confined to the central nervous system [26-28]. Here, these channels are protected from the binding of macrocyclic lactones by the highly effective P-gp mediated drug efflux at the blood-brain barrier which restricts drug penetration into the brain [21]. Therefore, and given the expression of a functionally active P-gp at the blood-brain barrier, macrocyclic lactones generally have a wide margin of safety in mammals at therapeutic doses [29].

## BRAIN PENETRATION OF MACROCYCLIC LACTONES IN P-GP DEFICIENT MICE

Several experimental models have been developed to analyse drug interactions with P-gp. *In vitro* models include the Caco-2 cell line, which shows, among numerous other carriers, a high expression of P-gp, and cell lines stably transfected with P-gp such as Madin-Darby canine kidney cells [7]. In these cellular systems interactions with P-gp have been demonstrated for a large number of drugs including ivermectin, selamectin, moxidectin, eprinomectin, abamectin and doramectin [30,31]. Furthermore, in 1994 a genetically engineered knockout mouse was established in which first only the *mdr1a* gene and later on both murine *mdr1* genes (*mdr1a* and *mdr1b*) were disrupted by insertional mutagenesis [32,33]. Despite the broad tissue expression of P-gp, loss of either or both genes did not result in an obvious phenotype or any physiological abnormality. The knockout mice were viable and fertile and almost indistinguishable from their wild-type littermates in a range of histological, hematological, serum-chemical and immunological parameters, but spontaneously develop colitis with age [33,34]. However, it has to be emphasised that laboratory mice grow up in a well-controlled and generally toxic-free environment where the importance of the protective function of P-gp may be less relevant. In these mice, the highly important role of P-gp for the safety of treatment with macrocyclic lactones was identified by serendipity. 

Following a mite infection of the generated *mdr1a* knockout mice, the mice were sprayed with a dilute solution of ivermectin which is routine in mite infections in an animal facility and is normally well tolerated by the mice even though they ingest part of the drug due to grooming activities. Following the ivermectin application, however, a number of *mdr1a(-/-)* knockout mice, but not the *mdr1a(+/+)* wild-type mice, died with paralytic symptoms including immobilization, inability to right themselves, recumbency, decreased breathing frequency, and finally, onset of a comatose state. After a more detailed toxicity analysis the researchers demonstrated that *mdr1a(-/-)* mice were 50- to 100-fold more sensitive to orally administered ivermectin (LD_50_ = 700-800 µg/kg in the knockout and 50-60 mg/kg in the wild-type mice) due to an increased accumulation in the brain [32,33]. These results were consistent with the suggested role of P-gp and the high expression in brain capillaries [16,17]. Application of radiolabelled ivermectin revealed that absolute brain concentrations were 87-fold higher in the brain of *mdr1a(-/-)* knockout mice compared with the wild-type mice (131 ± 16 ng/g *vs.* 1.5±1.2 ng/g), whereas the drug concentrations in most other tissues were only 3- to 4-fold higher. This general increase in tissue concentrations was likely due to an increased net uptake of ivermectin from the gastrointestinal tract combined with reduced elimination through the liver and kidney [32]. Even after intravenous and spot-on applications of 200 µg/kg ivermectin to *mdr1a,b(-/-)* knockout mice, where intestinal absorption does not affect the drug bioavailability, the absolute ivermectin concentrations in the brain were 59-fold (130 ng/g *vs.* 2 ng/g) and 49-fold (27 ng/g *vs.* 0.6 ng/g) higher in the knockout mice compared with the wild-type mice, respectively [35] Fig. (**2**). 

Apart from the genetically engineered *mdr1* knockout mice, researchers at the Merck Research Laboratories identified in the CF-1 mouse strain a subpopulation of mice which were much more sensitive to avermectins compared to other mice [36]. Further analysis revealed that these drug sensitive mice did not express the *mdr1a* P-gp in the brain capillary endothelial cells lining the blood-brain barrier due to an insertion of a solo long terminal repeat of the ecotropic murine leukemia virus, resulting in abnormal splicing of the *mdr1a* transcript and thereby leading to the translation of a non-functional P-gp [37,38]. As a consequence, high levels of ivermectin accumulated in the brain of the CF-1 mice, up to 70-fold, after oral drug application of 200 µg/kg [36,39]. 

Based on these data, it became absolutely clear that P-gp expression at the blood-brain barrier is the major and critical determinant for the safety margin of ivermectin and other macrocyclic lactones in mammals. These findings shed new light on clinical data from veterinary medicine that identified a subpopulation of Collie dogs as extremely sensitive to ivermectin in the early 1980s [40,41]. However, it has to be noted that P-gp at that time had not yet been localised in the blood-brain barrier and P-gp transport of macrocyclic lactones was completely unknown. Most interesting was a study by Pulliam *et al.* [42], demonstrating that ivermectin-sensitive Collie dogs showed highly increased ivermectin accumulation in the brain, suggesting that in ivermectin-sensitive dogs the protective barrier function of the blood-brain barrier is defective and ivermectin can penetrate into the brain unhindered Fig. (**2**). Referring to this study, Schinkel *et al.* [32] and other experts in the field hypothesized that ivermectin-sensitive Collies, analogous to the *mdr1 *knockout mice, must have a genetic deficiency in the canine *MDR1* gene resulting in the expression of a non-functional P-gp. From that point researchers began to clone and sequence the canine *MDR1* cDNA in order to identify the proposed genetic defect in ivermectin-sensitive Collies.

## IVERMECTIN-SENSITIVE COLLIES AND THE NT230(DEL4) *MDR1* MUTATION

When ivermectin-sensitive Collies are exposed to 100-120 µg/kg ivermectin they develop mild neurological toxicity including mydriasis, ataxia and depression [40,41], whereas much higher doses of up to 2 mg/kg are well tolerated by Beagle dogs or ivermectin non-sensitive Collies [29]. Ivermectin susceptibility, though, is not present in all individuals of the Collie breed and it is not related to sex, collie-eye anomaly, or hair coat type. Nevertheless, this was regarded as a genetically determined drug susceptibility [40,42,45]. In 2001, Mealey *et al.* [46] were the first to identify a 4-bp deletion mutation in the *MDR1* gene of an ivermectin-sensitive Collie. This nt230(del4) *MDR1* deletion produces a frame shift at amino acid position 75 followed by a premature stop codon at amino acid position 91 Fig. (**3**). This severely truncated protein is non-functional and was undetectable by Western blotting [47]. Based on microsatellite analyses it has been proposed that all dogs carrying this mutant nt230(del4) *MDR1* allele are descendants of a dog that lived in the United Kingdom in the 1800s, predating the emergence of formal breed lines [57]. 

Because of the predominant role of P-gp in drug disposition, mutation of the *MDR1* gene alters the pharmacokinetic properties of P-gp transported drugs, leading to enhanced oral bioavailability and reduced drug elimination through the liver, kidney and gut. Moreover, the brain penetration of P-gp transported drugs is increased and in many cases provokes neurological toxicity [8-10,48,49] Fig. (**1**). Clinical observations have already indicated that apart from macrocyclic lactones, the antidiarrheal drug loperamide, which is normally excluded from the brain by P-gp, causes neurological toxicosis in *MDR1(-/-)* dogs at normal therapeutic doses [50,51]. Although not explicitly analysed, loperamide toxicosis in these dogs was most likely due to increased brain penetration in the absence of P-gp [52,53]. An increased brain penetration of many further drugs was experimentally demonstrated in *mdr1* knockout mice including vinblastine, doxorubicin, paclitaxel, quinidine, ondansetron, cyclosporine and verapamil (see Table **1**). Although drug transport was not investigated with the canine P-gp for most of these drugs, increased brain penetration and central adverse effects also have to be assumed in *MDR1(-/-)* mutant dogs. The plasma pharmacokinetics of other drugs are altered in *MDR1* mutant dogs and so may provoke increased adverse effects in these dogs. For example, digoxin toxicity was documented in a *MDR1(-/-)* Collie dog which developed an unusually high serum digoxin concentration leading to digoxin toxicosis [54]. Furthermore, increased sensitivity to acepromazine and butorphanol was observed in *MDR1(-/-)* dogs that experienced a more pronounced and protracted central nervous system (CNS) depression compared to *MDR1* normal dogs [55]. A recent clinical study showed that the *MDR1* genotype is also highly relevant for veterinary oncology. Mealey *et al.* [56] analysed 34 dogs diagnosed with lymphoma that were to be treated with vincristine including four *MDR1(-/-)* and four *MDR1(+/-)* dogs. This study showed that *MDR1* mutant dogs are extremely susceptible to myelosuppression and gastrointestinal toxicosis induced by the vincristine treatment, resulting in higher morbidity and mortality rates and treatment delays [56]. 

Following identification of the nt230(del4) *MDR1* gene deletion mutation several *MDR1* genotyping methods were developed. Most of them use PCR amplification of the nt230 flanking region on exon 4 of the canine *MDR1* gene, followed by length polymorphism analysis using polyacrylamide gel electrophoresis [57-61]. More recently, a fluorogenic 5' nuclease TaqMan allelic discrimination method was described which allows *MDR1* genotyping without post-PCR processing and is useful for routine diagnostics [62]. To date, *MDR1* genotyping is also commercially available in many countries so that veterinarians can find the *MDR1* genotype in a canine patient before treatment is started with a P-gp transported drug.

### Breed Predisposition of the *MDR1* Mutation

Over the last few years, systematic genotyping analyses of the distribution of the nt230(del4) *MDR1* mutation among different breeds were performed on more than 15,000 dogs worldwide [76]. These studies revealed, that apart from the Collie, 12 further dog breeds are affected by this gene deletion mutation: Longhaired Whippet, Shetland Sheepdog, Miniature Australian Shepherd, Silken Windhound, McNab, Australian Shepherd, Wäller, White Swiss Shepherd, Old English Sheepdog, English Shepherd, German Shepherd and Border Collie (Table **2**) [57,61,76,77]. Among all of these dog breeds, the allelic frequency for the mutant *MDR1(-)* allele is highest in the Collie with similar frequency values worldwide: 51%-56% in the USA [57,61,78], 55%-59% in Germany [76,77], 60% in the United Kingdom [57] and 56% in Australia [79]. Apart from these purebred dogs, surprisingly high frequency values were also found in herding breed mixes as well as in many unclassified mixed breed dogs [61,76]. In contrast, several other dog breeds that also show close genetic relationships or share a breeding history with one of these predisposed dog breeds are presumed to be free of this mutation including the Bearded Collie, Anatolian Shepherd Dog, Greyhound, Belgian Tervuren, Kelpie, Borzoi, Australian Cattle Dog and Irish Wolfhound [57, 61,76].

Despite plenty of *MDR1* genotyping data, on a practical basis it is difficult for veterinarians and dog owners to recognise whether this *MDR1* gene deletion mutation is relevant for an individual canine patient/dog. Nevertheless, *MDR1* genotyping is essential for all of the purebred dogs listed in Table **2** and also for mixed breed dogs prior to extra-label use of higher doses of macrocyclic lactones, *e.g.* for the treatment of canine generalised demodicosis (see Table **3**), if severe and life-threatening adverse drug reactions are to be avoided. On the other hand, *MDR1* genotyping is not absolutely necessary in other purebred dog breeds not listed in Table **2**. 

## TREATMENT OF *MDR1* MUTANT DOGS WITH MACROCYCLIC LACTONES

Macrocyclic lactones have potent anthelmintic and ectoparasitic properties and are widely used in veterinary medicine for the treatment of parasitic diseases [81]. Commercially available products include the avermectins ivermectin, doramectin and selamectin as well as the milbemycins moxidectin and milbemycin oxime [82]. In vertebrates, all macrocyclic lactones are considered to have the same mechanism-based toxicity by binding to neuronal GABA-gated chloride channels [26-28]. Therefore, *MDR1* mutant dogs which do not express P-gp at the blood-brain barrier in general are prone to neurotoxicity by macrocyclic lactones due to the increased brain penetration. Nevertheless, treatment of these dogs with macrocyclic lactones does not inevitably result in neurological symptoms, since, the safety of treatment depends on the following four factors:

### Dosage/Treatment Indication

Neurotoxicosis is induced in *MDR1(-/-)* dogs after oral application of ( 100 µg/kg ivermectin or doramectin [40-42,60,83], ( 400 µg/kg moxidectin [58] or ( 5 mg/kg milbemycin oxime [84]. Treatment below these dosages, *e.g.* for heartworm prevention, is tolerated even by *MDR1* mutant dogs. The dosage of macrocyclic lactones is also crucial for the outcome from intoxication, irrespective of whether the macrocyclic lactone was therapeutically applied or accidentally ingested. For example, after the subcutaneous application of doramectin at 600 µg/kg two *MDR* mutant White Swiss Shepherd dogs showed severe neurotoxicosis which required intensive care, but both dogs fully recovered within 14 days [60]. In contrast, we attended two other clinical cases where an Australian Shepherd and a Collie dog, both with the *MDR1(-/-)* genotype, died within 5-6 days after application of a slightly higher dose of 1 mg/kg (unpublished cases). Both dogs showed extremely high drug concentrations in their brain see Fig. (**2**). 

### Route of Application

Spot-on applications of ivermectin and moxidectin did not induce neurological toxicity in *MDR1(-/-) *dogs at dosage of up to 1.0 mg/kg and 2.5 mg/kg, respectively [85,86]. In contrast, oral application of adequate doses for treatment purposes would be highly toxic for *MDR1* mutant dogs. Therefore, the route of application is crucial for the safety of treatment with macrocyclic lactones [87]. This particularly applies to spot-on formulations of moxidectin and selamectin which are labelled for use in dogs against a number of endo- and ectoparasitic diseases and should not be applied orally to *MDR1(-/-)* dogs. Ivermectin, apart from the oral application, is often extra-label used subcutaneously at concentrations of 400 µg/kg for the treatment of mange disease. This route of application in *MDR1(-/-)* dogs generally results in less severe neurotoxicosis which is, however, longer lasting compared with the oral route of application [60,88,89].

### Individual Compound

In dogs with homozygous *MDR1(-/-)* mutation, ivermectin and doramectin seem to have a similar neurotoxicological potential and induce severe neurological toxicity at dosages of 200-600 µg/kg which often results in coma and death of the animals [40,42,60]. In contrast, other macrocyclic lactones, such as selamectin, moxidectin and milbemycin oxime, claim to be safer in the treatment of *MDR1(-/-)* dogs and seem to have a lower neurotoxicological potential. Milbemycin oxime and selamectin only showed mild neurological toxicity in *MDR1* mutant dogs at oral doses of ≥ 5 mg/kg and >15 mg/kg, respectively [84,90,91]. Moxidectin seems to be intermediate in this respect and induced mild neurotoxicosis in *MDR1* mutant dogs at doses of ≥ 400-1000 µg/kg [58,92]. Nevertheless, it has to be emphasised that for these compounds the safety margin is also dramatically reduced if P-gp is not expressed at the blood-brain barrier, so that treatment of *MDR1* mutant dogs with macrocyclic lactones in general requires particular caution. 

### Heterozygous *MDR1(+/-)* or Homozygous *MDR1(-/-)* Genotype of the Dog

Although systematic studies on the application of macrocyclic lactones to *MDR1(-/-)* and *MDR1(+/-)* dogs have not been performed for any compound, many clinical cases of macrocyclic lactone intoxication in Collies or related dog breeds with unknown genetic *MDR1* status resulted in two different kinds of toxic reactions: dogs with mild ataxia and CNS depression or even more severe neurotoxicosis which quickly recovered (presumably *MDR1(+/-)* dogs) and dogs with severe and long-lasting intoxications (presumably or post-case documented *MDR1(-/-)* dogs) [51,83,93]. Furthermore, it was shown that *MDR1(+/+)* as well as *MDR1(+/-)* dogs can tolerate oral doses of ivermectin at up to 600 µg/kg [40,46,94], whereas this dosage would induce life-threatening neurotoxicosis in *MDR1(-/-)* dogs [42,88]. Therefore, *MDR1(+/-)* dogs can be regarded as having an intermediate macrocyclic lactone sensitive phenotype which is relevant in cases of a high dose protocol, *e.g.* for the treatment of canine generalised demodicosis [94]. However, it is unlikely (and the authors were not aware of any clinical case) that *MDR1(+/-)* dogs would suffer from coma or death even under such high therapeutic dosage regimens, unlike *MDR1(-/-)* dogs (see Table **3**).

## HEARTWORM PREVENTION IN DOGS WITH MACROCYCLIC LACTONES

Among the available macrocyclic lactones, four compounds are currently used as heartworm preventatives in a variety of different formulations: ivermectin, selamectin, milbemycin oxime and moxidectin. These compounds interrupt larval development during the first two months after infection and, therefore, have a long application phase and are administered monthly or even less frequently [95].

### Ivermectin

is marketed as a once monthly heartworm preventative at 6-12 µg/kg (*Heartgard^®^*) and also is microfilaricidal at this dosage [96]. Although *MDR1(-/-) *dogs are extremely sensitive to ivermectin, it has to be emphasised that no adverse drug reactions have been identified at these low preventive doses of ivermectin. Even after oral application of the 10-fold dose of 60 µg/kg [83] no adverse drug reactions were observed in ivermectin-sensitive Collies. This indicates that low dose ivermectin is a safe heartworm preventative even in *MDR1* mutant dogs [83]. 

Compared with other macrocyclic lactone compounds **selamectin **is unique within its clinical spectrum and is available in a topical formulation (*Stronghold^®^, Revolution^®^*). Selamectin is applied at 6-12 mg/kg once per month and at this dosage is effective at preventing heartworm infections and is additionally indicated for flea infestation, sarcoptic mange, ear mites and even tick infestation [90,97]. The safety of selamectin treatment was specifically analysed in ivermectin-sensitive Collies and it produced no adverse drug reactions, even at supra-therapeutic doses [90,91]. 

### Milbemycin oxime

is, apart from other indications, an effective heartworm preventative at a minimum recommended oral dose of 0.5 mg/kg at monthly intervals (*Interceptor^®^, Milbemax^®^*) [98]. When doses of 0.5-2.5 mg/kg milbemycin oxime were orally applied to ivermectin-sensitive Collies, no clinical signs of neurotoxicosis were observed [84]. Therefore, this compound can be safely used for heartworm prevention in *MDR1(-/-)* dogs. At higher concentrations of 5-10 mg/kg, as well as at daily dosing protocols of 0.5-2.8 mg/kg, which are normally well tolerated in dogs, milbemycin oxime provoked neurological toxicity in *MDR1* mutant dogs including ataxia, salivation and depression [84,99].

### Moxidectin

 has been more recently marketed as a heartworm preventative (*ProHeart^®^*) and has been shown to be safe and effective at oral doses of 3 µg/kg given monthly [100]. A safety evaluation was specifically performed in ivermectin-sensitive Collies using an oral application of up to 90 µg/kg. Even at this 30-fold therapeutic dosage, moxidectin produced no neurotoxic adverse effects [92], indicating that moxidectin can be safely used as a heartworm preventative in *MDR1(-/-)*dogs. Apart from the oral formulation, moxidectin is also approved for heartworm prevention in a topical combination formulation of 10% w/v imidacloprid plus 2.5% w/v moxidectin (*Advocate^®^, Advantage multi^®^*) at a monthly application [101]. At the recommended therapeutic dosage, 2.5 mg/kg of moxidectin is applied per interval and this treatment has been shown to be well tolerated, even in ivermectin-sensitive Collies [86]. 

In summary, ivermectin, selamectin, moxidectin and milbemycin oxime are effective and safe drugs for heartworm prophylaxis with varying spectra and routes of application [95]. All of these drugs can be safely administered to *MDR1(-/-)* mutant dogs at the preventative dosage and by the correct application [102]. However, it has to be emphasised that at doses higher than those used for heartworm prevention, *MDR1* mutant dogs will experience neurological toxicity with any of the macrocyclic lactones in this category (see Table **3**).

## MANGE TREATMENT IN DOGS WITH MACROCYCLIC LACTONES

Generalised demodicosis caused by *Demodex canis* mites is one of the most common skin diseases in dogs and is commonly regarded as difficult to treat successfully. Only a very few drugs are approved for the treatment of canine generalised demodicosis, including amitraz, an alpha-adrenergic receptor agonist used as a dip, and the topical combination formulation of 10% w/v imidacloprid plus 2.5% w/v moxidectin marketed as *Advocate^®^* or *Advantage multi^®^*. Apart from these medications, other macrocyclic lactones, although not approved for this indication, are commonly used in the management of this disease including ivermectin, doramectin and milbemycin oxime [103,104]. For example, ivermectin is administered at oral daily doses of 300-600 µg/kg, doramectin at 400-600 µg/kg and milbemycin oxime at 0.5-2.8 mg/kg for the treatment of canine generalised demodicosis [103,105-109]. Due to the hypersensitivity of *MDR1* mutant dogs to macrocyclic lactones, these medications have not been officially approved for the treatment of canine generalised demodicosis. Nevertheless, several therapeutic protocols exist which are generally well tolerated in *MDR1* normal dogs. These protocols usually use a gradual increase in dose over the first few days of treatment in order to recognise sensitive dogs before reaching a critical dosage that would induce life-threatening intoxication. This procedure was essential before the nt230(del4) *MDR1* mutation was discovered, but nowadays it may be replaced by *MDR1* genotyping. Nevertheless, due to the widespread distribution of the *MDR1* mutation amongst different breeds, also including many mixed breed dogs, it is difficult to recognise whether an individual canine patient might be affected by this mutation or not. Therefore, the gradually increasing treatment protocol is still recommended. In detail, such protocols start with *e.g.* an oral ivermectin application at 50 µg/kg on the first day, followed by 100 µg/kg on the second day, 150 µg/kg on the third day, 200 µg/kg on the fourth day, and finally 300 µg/kg on the fifth and following days for at least 12 weeks [110]. During this dosage regimen it is very important to recognise the initial symptoms of ivermectin-induced neurological toxicity, such as ataxia, mydriasis and hypersalivation, as soon as possible and to immediately discontinue treatment in such a case.

### Ivermectin

As already mentioned above, ivermectin orally applied at 50-60 µg/kg is well tolerated by ivermectin-sensitive dogs [42,83], but neurological toxicity is induced at higher doses of >100 µg/kg: after application of 100-120 µg/kg, mild depression and ataxia, as well as disorientation and mydriasis have been observed within 12 hours after application [40,41,92,111]; 125-170 µg/kg induced more severe ataxia, stupor, recumbency, head bobbing, apparent blindness, facial twitches, hypersalivation, episodes of hyperventilation and bradycardia [40,41,83,112,113]; still higher doses of 200-250 µg/kg caused severe neurotoxicosis, including depression, ataxia and apparent blindness as early onset symptoms, as well as vomiting, paddling movements, tremor and excessive salivation, followed by stupor, feeble attempts to crawl, recumbency, and finally non-responsiveness and coma within 30-50 hours after application, often resulting in death [40-42,88,113-115]; very high doses of ivermectin, of up to 600 µg/kg, accelerated the onset of symptoms and often resulted in death within 48 hours or euthanasia if the option of mechanical ventilation was rejected [40,42,88]. In conclusion, treatment protocols with 300-600 µg/kg ivermectin or even doramectin are unfeasible in *MDR1* mutant dogs and would clearly result in life-threatening intoxication (see Tables **3** and **4**). 

In contrast, acute and subchronic ivermectin toxicity studies in *MDR1* normal dogs demonstrated a large safety margin: ivermectin can be administered to Beagles, as well as to ivermectin non-sensitive Collies, at a single oral dose of 2 mg/kg or at a daily oral dose of 500 µg/kg over 14 days without any evidence of toxicosis [29]. Only at higher doses of 1 mg/kg ivermectin applied daily for 14 weeks or at a single dose of 2.5 mg/kg did these dogs show mydriasis as the initial symptom of drug-induced neurological toxicity [116,117]. Even higher doses of 5-20 mg/kg additionally caused ataxia and tremor, and 40 mg/kg proved to be fatal [93,115,116]. The oral LD_50_ for ivermectin in Beagle dogs was estimated to 80 mg/kg. Post-mortem, these ivermectin-poisoned dogs were pathologically normal and no specific lesions were observed in the brain [118].

### Moxidectin

Several *in vitro* transport studies also confirmed that moxidectin is transported by P-gp, although it seems to be a weaker substrate and inhibitor of P-gp compared with ivermectin [30,119-121]. Closer analysis of the interaction between moxidectin and P-gp revealed that moxidectin can inhibit the P-gp efflux function with a similar efficiency compared to ivermectin, however, it requires concentrations 10 times higher to reach the same inhibitory effect. This would be consistent with a lower affinity binding of moxidectin to P-pg compared with other macrocyclic lactones [31]. Very recently, Kiki-Mvouaka *et al.* [44] analysed the *in vivo* pharmacokinetics of moxidectin in the *mdr1a,b(-/-)* knockout mouse model in comparison with ivermectin. After subcutaneous applications of 200 µg/kg for both drugs, equal drug concentrations of approximately 65 ng/g were found in the brain 24 hours after application, indicating that in the absence of P-gp in the blood-brain barrier both compounds show comparable brain penetration. However, in the wild-type mice absolute brain concentrations were more than 10-fold higher for moxidectin, demonstrating that P-gp *in vivo* transports moxidectin less effectively at the blood-brain barrier compared with ivermectin [44] see Fig. (**2**). 

For the treatment of canine generalised demodicosis, 200-400 µg/kg moxidectin is commonly applied orally per day and this treatment is normally well tolerated in dogs [108]. Higher doses of 1 mg/kg were even tolerated in Beagle dogs with no clinical signs of neurological toxicity [122]. As already mentioned above, moxidectin is safely tolerated even by ivermectin-sensitive Collies, but only at low oral doses of 90 µg/kg [83,92]. However, as shown in an Australian Shepherd with the *MDR1(-/-)* genotype, which was treated with a gradually increasing dosage protocol, neurological toxicity was induced after reaching the target dose of 400 µg/kg. After discontinuing the treatment the dog fully recovered, indicating that the neurotoxicosis was induced by moxidectin [58] (Table **4**). Therefore, a treatment protocol of 400 µg/kg moxidectin orally per day cannot be applied to *MDR1(-/-)* dogs for the treatment of canine generalised demodicosis. On the other hand and in contrast to this extra-label oral application, moxidectin is approved for the treatment of canine generalised demodicosis and a range of other endo- and extoparasites in a topical combination formulation of 10% w/v imidacloprid plus 2.5% w/v moxidectin [123]. To the best of our knowledge, this topical formulation currently represent the only macrocyclic lactone-containing treatment option which is licensed for the treatment of canine generalised demodicosis. The safety of this formulation was evaluated under field conditions in different dog breeds and was generally well tolerated at the therapeutic dosage [104,123]. During the safety evaluation this combination formulation was also specifically administered to ivermectin-sensitive Collies. At the approved topical application, this formulation was safely tolerated by *MDR1* mutant Collies even at the 5-fold therapeutic dosage containing 32.5 mg/kg moxidectin [86]. However, after oral application of only 40% (1 mg/kg) of the recommended topical dose, neurological toxicity occurred in the drug-sensitive Collies [124], emphasising that oral ingestion of this formulation must definitely be precluded in *MDR1(-/-)* dogs.

### Milbemycin Oxime

Milbemycin oxime has been used for the treatment of generalised demodicosis at doses ranging from 0.5-2.8 mg/kg [103,109]. Only one study is available which analysed the safety of milbemycin oxime in ivermectin-sensitive Collies. In this study, the characteristic neurological toxicity, albeit of short duration, was observed in individual Collies applied with 5 mg/kg milbemycin oxime orally, and included mild depression, excessive salivation and ataxia [84]. At the higher dosage of 10 mg/kg all sensitive Collies developed signs of mild depression and ataxia within 6 hours after treatment. Neurotoxicosis persisted for at least 24 hours, but all dogs fully recovered within 48 hours after treatment. In another study, milbemycin oxime was applied to two ivermectin-sensitive Collies as a sesame oil solution in gelatin capsules. Whereas at 1.25 mg/kg no adverse drug reactions were observed, both dogs became ataxic within 4 hours of treatment at 2.5 mg/kg [90]. In contrast, in an earlier study of the application of milbemycin oxime at dosages of up to 25 mg/kg in rough-coated Collies, no adverse drug reactions were induced [125]. However, it has to be assumed that these dogs, although of the Collie breed, were not affected by the *MDR1* mutation. More recently, Barbet *et al.* [99] analysed the safety of treatment with milbemycin oxime in 22 dogs diagnosed with generalised demodicosis including two *MDR1(-/-)* and one *MDR1(+/-)* dog. All dogs received milbemycin oxime at a daily dose of 1-2.2 mg/kg, which normally is well tolerated in dogs [109]. None of the *MDR1* normal dogs nor the heterozygous *MDR1(+/-)* dogs experienced any adverse drug reactions under treatment. In contrast, and despite the low dose initiation of treatment with 300-800 µg/kg/day, both *MDR1(-/-)* mutant dogs experienced ataxia following an increase of the dose to 1.5-1.6 mg/kg/day. When the treatment dose of milbemycin oxime then was decreased to a tolerable dose of 600 µg/kg, both dogs recovered [99] (Table **4**). This study clearly indicates that the knowledge of the *MDR1* genotype is critical in order to achieve a milbemycin oxime dosage regimen that is tolerated without causing adverse drug reactions. It further shows that milbemycin oxime may be the safer choice than ivermectin or doramectin for the treatment of generalised demodicosis in *MDR1(-/-)* dogs.

### Selamectin

Selamectin is marketed as a spot-on formulation with a minimum therapeutic dosage of 6 mg/kg (*Stronghold^®^, Revolution^®^*). Although not effective for the treatment of canine generalised demodicosis, selamectin shows activity against both insect and arachnid classes of ectoparasites and is licensed for the control of canine sarcoptic mange [90,97,126]. *In vitro* transport studies showed that selamectin is transported by P-gp as equally as ivermectin [30,121]. Thus, it was assumed that *MDR1* mutant dogs would also exhibit increased drug sensitivity against selamectin. Indeed, an *in vivo* study by our laboratory confirmed that selamectin at concentrations of 12 mg/kg (representing the maximum dose for the body weight range) shows significantly higher brain penetration in *mdr1a,b(-/-)* knockout mice compared with wild-type mice by any route of application (oral, intravenous, spot-on) [35]. However, the brain concentration ratios (knockout *vs.* wild-type mice) of 5-fold to 10-fold were much less pronounced than the respective ratios for ivermectin applied at 200 µg/kg (i.e. 36-fold to 60-fold) see Fig. (**2**). Furthermore, brain-to-plasma concentration ratios in the wild-type mice were much higher for selamectin than for ivermectin (0.32 *vs.* 0.09, respectively), which is consistent with a higher efflux rate at the blood-brain barrier for ivermectin than for selamectin [35]. These findings support more recent data from *in vitro* studies by Lespine *et al.* [31] which showed that ivermectin has much higher affinity for P-gp compared with selamectin (*K*_i_ values: 0.05 µM *vs.* 1.0 µM, respectively). Based on this data, it may be speculated that the dimensions of the sugar moiety on the macrocycle (disaccharide substitution in ivermectin, monosaccharide substitution in selamectin) account for these different affinities to P-gp and can thus determine the extent of brain penetration of the respective compounds. 

During a safety evaluation of the topical selamectin formulation, application studies were specifically performed in ivermectin-sensitive Collies. Although increased brain penetration has to be assumed in these dogs, selamectin produced no adverse drug reactions at a dosage of 40 mg/kg topically or 15 mg/kg orally, which represent 3-7 times the minimum therapeutic dosage of 6 mg/kg [90,91] (Table **4**). Thus, the neurotoxic potential of selamectin seems to be much lower compared to all of the other macrocyclic lactones which provoke neurological toxicity in *MDR1* mutant dogs at much lower dosages and which reach much lower drug concentrations in the brain see Fig. (**2**). Therefore, we would anticipate that selamectin and ivermectin would exhibit different affinities or different intrinsic activities for vertebrate GABA-activated chloride channels. To prove this conjecture, it will be necessary to conduct comparative receptor binding assays in brain preparations. These should specifically address the role of the substitutions at positions C5 (NOH in selamectin and OH in ivermectin) and C25 (cyclohexyl in selamectin and *sec*-butyl/isopropyl in ivermectin B1) since these have previously been reported to significantly affect the antiparasitic activity of both compounds [81,127,128].

## *MDR1* SINGLE NUCLEOTIDE POLYMORPHISMS AND IVERMECTIN SENSITIVITY

Several clinical studies showed that ivermectin and even milbemycin oxime or moxidectin applied at a high dose protocol for the treatment of canine generalised demodicosis are generally well tolerated in *MDR1* normal dogs [103]. However, signs of subchronic neurotoxicity are occasionally seen in individual dogs treated with a daily high dose protocol. In these dogs, the onset of toxicity signs is seen several days or weeks after initiation of the treatment, in particular with ivermectin at 400-600 µg/kg/day, but these normally resolve after discontinuing the treatment, indicating that the macrocyclic lactone causes this intoxication [94]. These subchronic neurotoxicity reactions most likely represent individual differences in the sensitivity to ivermectin based on genetic variants [110]. For example, Bissonnette *et al.* [94] described a *MDR1(+/-)* juvenile mixed breed dog which showed neurological toxicity including ataxia, tremor and depression after daily ivermectin application at 670 µg/kg after seven weeks of therapy, indicating that at least one intact *MDR1* allele may protect the dog from acute neurotoxicity after high dose applications of ivermectin. On the other hand, dogs that actually have the intact *MDR1(+/+)* genotype, developed subchronic neurological toxicity at much shorter treatment intervals. It may be speculated that these dogs show lower expression of P-gp at the blood-brain barrier or that they may express a less active polymorphic P-gp. Currently, more than 30 single nucleotide polymorphisms are known in the canine *MDR1* gene (see Table **5**) which might affect the transport function of P-gp, such as the Gln532Arg amino acid substitution which is located in direct proximity to the highly conserved and functionally important ABC signature motif of P-gp. However, whether one of these polymorphisms indeed correlates with increased drug sensitivity under high dose treatment protocols with macrocyclic lactones has to be investigated further.

## INTOXICATIONS FROM HORSE DEWORMING MEDICATION

Ivermectin and moxidectin are commonly used in horses to treat parasitic diseases and are available as oral paste, oral liquid gel or tablet formulations, usually with 12-23 mg/g of the active drug. As these preparations deliver doses intended for the treatment of horses, they are highly concentrated and contain very high absolute amounts of drug (≥ 120 mg ivermectin or > 200 mg moxidectin per applicator or package) which result in severe intoxication when accidentally ingested by dogs [130-134], particularly when the dog has the *MDR1(-/-)* genotype [135-137]. The severity of ivermectin or moxidectin induced neurotoxicosis in such cases is generally a dose-dependent phenomenon. Therefore, prognosis and successful treatment can only be predicted on the basis of certain knowledge about the amount of drug ingested, although in most cases this cannot be reconstructed. In addition, the prognosis and eventual outcome depend on a number of further factors, including, first of all, the *MDR1* genotype of the dog, the individual as well as breed-typical constitution of the dog (which is somewhat different *e.g.* between Collie and Longhaired Whippet dogs) and the history of the toxicosis development (how rapidly and with which clinical signs it appears). Generally, severe clinical CNS depression occurring within 1-2 hours after ingestion or rapidly worsening toxicosis indicate the presence of the *MDR1* mutation and/or ingestion of very high doses of ivermectin. Mostly, these cases have a more critical progression and worse prognosis. On the other hand, *MDR1* mutant dogs with a slow onset of clinical signs, typically mydriasis, ataxia and apparent blindness within 4-8 hours after ingestion, have normally ingested low amounts and can be given a good prognosis. Although the recovery of poisoned dogs may take several weeks, many cases with long comatose episodes have returned to complete health [40,41,88,113,137]. This, however, requires good nursing and supportive care, including fluid treatment, nutritional support and vigilant cardiopulmonary monitoring.

## TREATMENT OF MACROCYCLIC LACTONE INDUCED NEUROTOXICOSIS

Currently, there is no specific and safe antidote available for the treatment of macrocyclic lactone-induced toxicosis. Therefore, treatment is solely based on symptomatic and supportive care [138]. Following oral ingestion, the initial therapy should focus on drug removal by inducing emesis as soon as possible, or by gastric lavage. Then the serial administration of adsorbents (*e.g.* activated charcoal) is indicated, as long as the dog shows responsiveness, in order to discourage enteral absorption of the parent compound and to enhance the elimination of ivermectin via the faeces, which is the main route of excretion [139]. In contrast, forced diuresis will not facilitate the excretion of ivermectin. During long lasting phases of nonresponsiveness and recumbency, supportive care, parenteral alimentation and prevention of decubital ulcers are of particular importance, while electrolytes, fluid balance, blood pressure, heart rate, body temperature, blood gases and respiratory function have to be monitored [40]. In order to reduce gastric irritation and acidification overshoot, substances which inhibit gastric acid secretion such as cimetidine have been applied [118]. However, as cimetidine is a substrate of P-gp, omeprazole is recommended for better medication in *MDR1* mutant dogs. In severe cases with pronounced respiratory depression, mechanical ventilation may be required. 

In some cases of severe neurotoxicosis where the dogs became unresponsive, physostigmine (a cholinesterase inhibitor) was administered slowly twice a day at a total dose of 40 µg/kg intravenously [41,89,113]. Physostigmine increases the synaptic concentration of acetylcholine in the central and peripheral nervous system. In the central nervous system acetylcholine acts as an excitatory neurotransmitter and induces central stimulation. In the periphery, acetylcholine acts parasympathomimetic and might be beneficial for gastrointestinal and motor stimulation. In the unresponsive dogs, physostigmine application resulted in a transient increase of responsiveness, muscle activity, heart and respiratory rate, and attempts to drink and eat, whereas in mild intoxication and responsive dogs it was of little benefit [41]. Nevertheless, in our experience physostigmine application is a helpful premedication in the recovery phase before food is made available or the dog is exercised by assisted walking. However, the duration of action for physostigmine lasted only 30-90 minutes. Physostigmine has no potency in accelerating the recovery of the dogs, or for general improvements of the outcome. Furthermore, in cases of overdose, physostigmine may be associated with the development of convulsions and bradycardia, and therefore must be handled with caution [41]. Treatment with glycopyrrolate before physostigmine administration may be warranted to avoid severe side effects, *e.g.* bradycardia. In summary, physostigmine is beneficial for transiently vitalizing a dog's constitution and for encouraging the owners that recovery might be possible. The major difficulty, however, involves the potential toxicity of physostigmine itself.

Picrotoxin has been used in a few cases of ivermectin-induced neurotoxicosis since it blocks GABA-activated chloride channels, and therefore was suggested as a possible antidote to ivermectin poisoning [140]. It was given by intravenous infusion at a dosage rate of 1 mg/min for 8 minutes and appeared to reverse severe CNS depression. However, picrotoxin infusion also induced violent clonic seizures 30 minutes after application which required treatment with thiopental. Because of this, and due to its narrow safety margin, picrotoxin cannot be recommended for routine use as an antidote for ivermectin intoxication [140].

In the case of ivermectin-induced tremor, which often occurs as a late-onset symptom (typically within 12 hours after oral drug ingestion), benzodiazepine drugs such as diazepam should be avoided as avermectins enhance binding of these drugs to the GABA receptor and further enhance the GABAergic activity, leading to a more pronounced CNS depression [139]. Although not approved in a large number of dogs, propofol as a short acting hypnotic drug might be an appropriate medication in this phase of neurotoxicosis [132, 133].

Although there has been intensive research on macrocyclic lactones over three decades, the search for an effective and direct antidote against macrocyclic lactone-induced neurotoxicosis has not yet been successful. Therapy of intoxication is still based on symptomatic relief, but it lacks a macrocyclic lactone receptor binding antagonist. Ideally, this antagonist should exhibit high binding affinity without or with much less intrinsic activity than the macrocyclic lactones used therapeutically. 

## CONCLUSIONS

Identification of the nt230(del4) *MDR1* mutation in drug-sensitive dogs has clearly improved the safety of treatment of parasitic diseases with macrocyclic lactones. Pharmacogenetic diagnostics can determine the *MDR1* genotype for an individual canine patient before macrocyclic lactone treatment is started which is particularly important for dog breeds highly predisposed towards this mutation. Depending on the *MDR1* genotype predictions can be made on whether the treatment will be safe and beneficial or whether the dog may experience neurological toxicity following drug application. 

## Figures and Tables

**Fig. (1):**
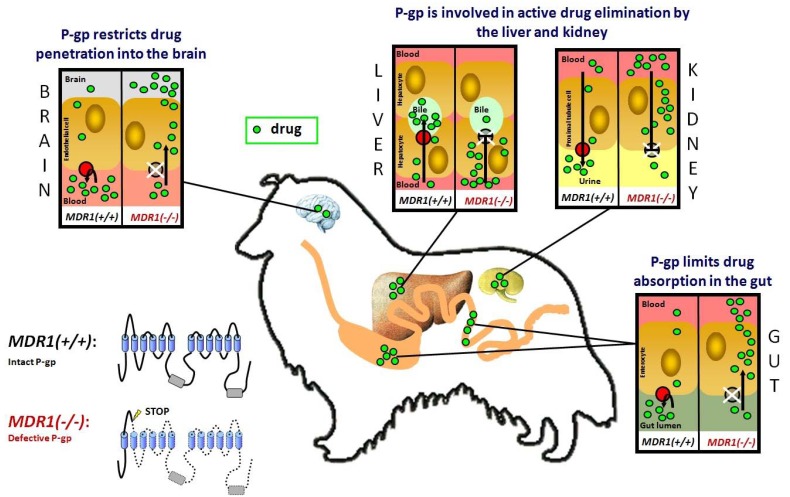
The role of P-gp in drug disposition. P-glycoprotein (shown in red) is an ATP-driven efflux transporter which pumps its substrates out of the cell. The intact P-gp limits drug entry into the organism after oral administration, promotes drug elimination into bile and urine, and restricts drug penetration across the blood-brain barrier. In *MDR1(-/-)* dogs which do not express a functional P-gp, enteral drug absorption is enhanced, biliary and urinary drug elimination is reduced, and the permeation of blood-tissue barriers is increased at the blood-brain barrier, blood-testis barrier and blood-placenta barrier. As a consequence, P-gp transported drugs can cause an increase in adverse effects in these dogs. This particularly applies to macrocyclic lactones, which would normally be efficiently transported by P-gp.

**Fig. (2):**
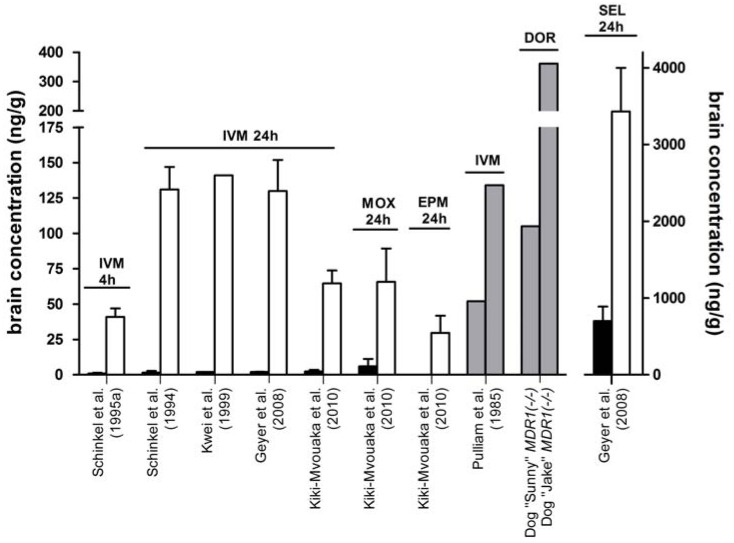
Brain penetration of macrocyclic lactones in wild-type mice (black columns), as well as in P-gp deficient mice (white columns) and dogs (grey columns). Ivermectin (IVM), moxidectin (MOX), eprinomectin (EPM), doramectin (DOR) and selamectin (SEL) were experimentally given to *mdr1a(-/-)* knockout mice (Schinkel *et al*. 1995a [43], 1994 [32]), *mdr1a,b(-/-)* double knockout mice (Geyer *et al*. 2008 [35], Kiki-Mvouaka *et al*. 2010 [44]), drug-sensitive CF-1 mice (Kwei *et al*. 1999 [39]), and ivermectin-sensitive Collies (Pulliam *et al*. 1985 [42]) or were therapeutically applied to *MDR1(-/-)* dogs at the following dosages: 200 µg/kg orally [32,35,39,42 left column,^43^], 200 µg/kg subcutaneously [44], 600 µg/kg orally [42 right column] and 1 mg/kg doramectin subcutaneously. Absolute drug concentrations in brain tissue were determined by liquid scintillation counting using the respective radiolabeled drugs [32,35,39,43] or by HPLC analysis [42,44]. Generally, drug concentrations in the brain were marginal in the wild-type mice and dramatically increased in the absence of P-gp. The two *MDR1(-/-)* dogs, "*Sunny*" and "*Jake*", were therapeutically given 1 mg/kg doramectin and developed severe neurotoxicosis. Both dogs died 5-6 days after treatment and were subjected to necropsy within 18 hours of death.

**Fig. (3):**
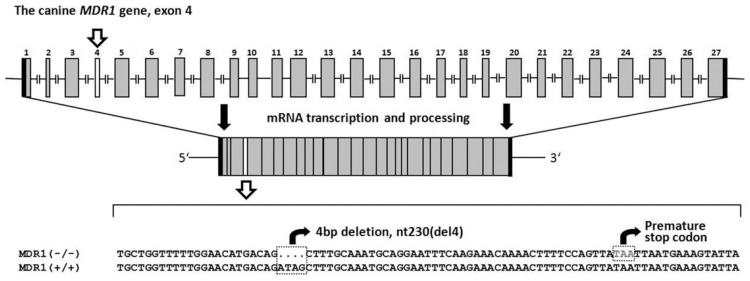
The nt230(del4) MDR1 mutation in dogs. The canine *MDR*1 gene is organised in 27 exons, located on chromosome 14. The transcribed *MDR1* coding sequence comprises 3846 bp and codes for the 1281 amino acids canine P-gp. The nt230(del4) mutation was localised to exon 4 and involves a 4-bp deletion followed by a premature stop codon. The truncated P-gp expressed from the mutant *MDR1(-)* allele is non-functional. Dogs with a homozygous nt230(del4) mutation are extremely sensitive to macrocyclic lactones.

**Table 1.** Drugs Transported by P-gp which Show Enhanced Brain Concentrations in *mdr1a(-/-)* or *mdr1a,b(-/-)* Knockout Mice Compared to Wild-Type Mice after Intravenous Application (if not Otherwise Stated). Macrocyclic Lactones are Depicted in Bold Face

Drug	Time after Application	Brain Concentration Ratio [Knockout / Wild-Type]	Reference
**Ivermectin**	24 h	87^a^,^c^	[32]
**Ivermectin**	24 h	60^b^,^c^	[35]
**Ivermectin**	4 h	46^a^,^c^	[43]
**Ivermectin**	8 h	36^b^	[35]
Nelfinavir	4 h	36^a^	[63]
Digoxin	4 h	35^a^	[64]
Tacrolimus	5 h	33^a^	[65]
Quinidine	0.5-5 h	33^b^,^d^	[53]
Quinidine	10 min	28^a^,^e^	[66]
**Ivermectin**	24 h	27^b^,^d^	[44]
Flesinoxan	3 h	27^a^	[67]
Vinblastine	4 h	22^a^	[32]
Verapamil	0.5-5 h	21^b^,^d^	[53]
Amiodarone	0.5-5 h	19^b^,^d^	[53]
Cyclosporin A	4 h	17^a^	[64]
Loperamide	0.5-5 h	17^b^,^d^	[53]
Loperamide	4 h	14^a^,^c^	[52]
Paclitaxel	24 h	12^b^	[68]
**Moxidectin**	24 h	11^b^,^d^	[44]
Indinavir	4 h	11^a^	[63]
Verapamil	1 h	9.5^a^	[69]
Asimadoline	1 h	9.1^b^	[70]
Metoclopramide	0.5-5 h	7.6^b^,^d^	[53]
Saquinavir	4 h	7.4^a^	[63]
Docetaxel	24 h	6.2^b^	[71]
**Selamectin**	24 h	5.0^b^,^c^	[35]
Doxorubicin	24 h	5.0^a^	[72]
Cortisol	2 h	4.6^b^,^d^	[73]
Ondansetron	30 min	4.0^a^	[52]
Sparfloxacin	2 h	3.9^b^,^e^	[74]
Doxorubicin	1 h	3.2^a^,^e^	[75]
Grepafloxacin	2 h	2.9^b^,^e^	[74]
Dexamethasone	4 h	2.5^a^	[64]
Morphine	4 h	1.7^a^	[64]

a *mdr1a*(-/-) knockout mice were used

b *mdr1a,b*(-/-) double knockout mice were used

c oral application

d subcutaneous injection

e data represent brain-to-plasma partition coefficient (K_p,brain,ko_ / K_p,brain,wt_).

**Table 2.** Breed Distribution of the nt230(del4) *MDR1* Mutation in Dogs Worldwide

Dog Breed	Range of Allelic Frequency (%) *MDR1(-)*	References
Collie	55 – 57	[57,61,76-79]
Longhaired Whippet	42	[57]
Shetland Sheepdog	7– 35	[57,61,76,77,80]
Miniature Australian Shepherd	20 – 26	[57,61]
Silken Windhound	18	[57]
McNab	17	[57]
Australian Shepherd	17 – 46	[57,61,76,77]
Wäller	17 – 19	[76,77]
White Swiss Shepherd	14	[76]
Old English Sheepdog	1 – 11	[57,61,76,80]
English Shepherd	7	[57]
German Shepherd	6	[61]
Border Collie	1 – 2	[61,76,77,80]
Herding-breed mix	6 – 7	[61,76]
Mixed breed	2 – 7	[61,76]

Note: Data from the referenced studies were included when at least 30 dogs were analysed per breed.

**Table 3.** Treatment Safety of Ectoparasitic and Endoparasitic Infections with Macrocyclic Lactones in *MDR1*(+/+) Normal and *MDR1*(-/-) Mutant Dogs

Indication	Drug	Dosage	Label	* MDR1(+/+)*	* MDR(-/-)*
Heartworm prevention	Ivermectin	6-12 µg/kg PO once monthly	Heartgard^a^	+	+
	Moxidectin	170 µg/kg SC every six months	ProHeart^a^	+	+
	Moxidectin	2.5 mg/kg moxidectin + 10 mg/kg imidacloprid spot-on monthly	Advocate^b^, Advantage multi^a^	+	+
	Selamectin	6 mg/kg spot-on monthly	Stronghold^b^, Revolution^a^	+	+
	Milbemycin oxime	500 µg/kg milbemycin oxime + 5 mg/kg praziquantel PO monthly	Milbemax^b^	+	+
	Milbemycin oxime	500-990 µg/kg milbemycin oxime PO monthly	Interceptor^a^	+	+
Generalised demodicosis	Moxidectin	2.5 mg/kg moxidectin + 10 mg/kg imidacloprid spot-on monthly	Advocate^b^, Advantage multi^a^	+	+
	Moxidectin	200-400 µg/kg PO daily	Extra-label	+	-
	Ivermectin	400-600 µg/kg PO daily	Extra-label	+	-
	Doramectin	600 µg/kg SC weekly	Extra-label	+	-
	Milbemycin oxime	0.5-2.0 mg/kg PO daily	Extra-label	+	-
Other ectoparasitic and endoparasitic infections	Ivermectin	50-200 µg/kg PO once	Extra-label	+	+/-c
	Ivermectin	300-400 µg/kg PO or SC weekly	Extra-label	+	-
	Moxidectin	250 µg/kg SC weekly	Extra-label	+	?
	Moxidectin	400 µg/kg PO every 3-4 days for 3-6 weeks	Extra-label	+	-
	Moxidectin	2.5 mg/kg moxidectin + 10 mg/kg imidacloprid spot-on monthly	Advocate^b^, Advantage multi^a^	+	+
	Selamectin	6 mg/kg spot-on monthly	Stronghold^b^, Revolution^a^	+	+
	Milbemycin oxime	500 µg/kg milbemycin oxime + 5 mg/kg praziquantel PO monthly	Milbemax^b^	+	+
	Milbemycin oxime	500-990 µg/kg milbemycin oxime PO monthly	Interceptor^a^	+	+

a FDA approved

b EMEA approved

c toxic at > 100 µg/kg; PO, oral application, SC, subcutaneous application; "+", tolerated, "-", not tolerated, may induce neurotoxicosis.

**Table 4.** Neurotoxic Potential of Macrocyclic Lactones and Treatment Outcome in MDR1 Mutant Dog

Compound	Breed (Genotype or Phenotype)*	Dose (Application)	Clinical Signs of Neurotoxicosis and Outcome	Reference
Ivermectin	Collie (ISC)	60 µg/kg (PO)	No	[83]

	Collie (ISC)	100-120 µg/kg (PO)	Mild depression, ataxia, disorientation, mydriasis, recovery	[40,41,92,111]
	Collie (ISC)	125-170 µg/kg	Ataxia, recumbency, stupor, apparent blindness, hypersalivation, recovery	[83,112,113]
	Collie (ISC)	200-250 µg/kg (PO)	Ataxia, depression, apparent blindness, paddling movements, tremor, excessive salivation, stupor, coma, death/recovery	[40,42,88,113-115]
	Collie (ISC), Australian Shepherd *MDR1(-/-)*	200 µg/kg (SC)	Ataxia, loss of vision, hypersalivation, recumbency, stupor, recovery	[88,89]
	Collie *MDR1(-/-)*	400 µg/kg (SC)	Ataxia, salivation, tremor, nonresponsiveness, stupor, coma, recovery	AUD

	Collie (ISC)	1 mg/kg (spot-on)	No	[85]
Doramectin	Collie	200 µg/kg (SC)	Apparent blindness, ataxia, hypersalivation, recumbency, recovery	[129]
	2 White Swiss Shepherd dogs *MDR1(-/-)*	700 µg/kg (SC)	Loss of vision, ataxia, depression, hypersalivation, hyperventilation, tremor, recumbency, recovery	[60]
	Collie *MDR1(-/-)*, Australian Shepherd * MDR1(-/-)*	1 mg/kg (SC)	Ataxia, tremor, stupor, coma, death	AUD

Selamectin	Collie (ISC)	40 mg/kg (spot-on) 15 mg/kg (PO)	No	[90,91]
Moxidectin	Collie (ISC)	90 µg/kg (PO)	No	[92]
	Australian Shepherd *MDR1(-/-)*	100 µg/kg increased to 400 µg/kg (PO)	Ataxia, crawling, hyperexcitability, recovery	[58]
	Collie (ISC)	32.5 mg/kg (plus 130 mg/kg imidacloprid) (spot-on)	No	[86]

Milbemycin oxime	Collie *MDR1(-/-)*	800 µg/kg/day incrementally increased to 1.5 mg/kg/day (PO)	Ataxia, recovery	[99]
		300 µg/kg/day incrementally increased to 1.6 mg/kg/day (PO)	Ataxia, recovery	
	Collie (ISC)	1.25 mg/kg (PO) 2.5 mg/kg (PO)	No Ataxia, recovery	[90]
	Collie (ISC)	2.5 mg/kg (PO) 5 mg/kg (PO)	No Ataxia, salivation, depression, recovery	[84]

* Before the discovery of the nt230(del4) MDR1 mutation, ivermectin-sensitive Collies (ISC) were identified by test application of 120-200 µg/kg ivermectin orally followed by documentation of neurological toxicity including ataxia and CNS depression. PO, oral application; SC, subcutaneous application; AUD, author's unpublished data

**Table 5.** Single Nucleotide Polymorphisms in the Canine *MDR1* cDNA Sequence

Single Nucleotide Polymorphism	Exon	GenBank Accession No.	Amino Acid Substitution	PolyPhen Prediction	SIFT Prediction
A23G	2	AJ419568	Silent		
A51G	2	AJ419568	Silent		
A86G	3	AJ419568	Silent		
A265G	4	AF536758, FJ617477	Thr89Ala	Benign	Tolerated
T564C	7	AJ419568	Silent		
A574G	7	AF045016	Silent		
A591C	7	AF536758	Silent		
G635C	7	AF045016	Silent		
A862G	9	AF536758	Arg288Gly	Benign	Tolerated
T985A	9	AF045016	Ser329Thr	Benign	Tolerated
A996G	9	AF045016	Silent		
T1232C	12	AJ419568	Silent		
A1595G	14	AB066299, AF045016	Gln532Arg	Probably damaging	May affect protein function
G1863A	15	AF092810	Silent		
G1914C	16	AF092810	Glu638Asp	Benign	Tolerated
A2082T	17	AF045016	Silent		
C2086T	17	AB066299, AF045016	Pro696Ser	Benign	Tolerated
A2181G	17	AF092810	Silent		
A2258T	18	AF092810	Asn753Ile	Benign	May affect protein function
C2322T	18	AF092810	Silent		
C2328T	19	AF092810	Silent		
G2349A	19	AF092810	Silent		
C2426T	20	AF092810	Pro809Leu	Possibly damaging	Tolerated
A2451C	20	AF092810	Silent		
G2471T	20	AF092810	Silent		
A2601G	21	AY582533	Silent		
G2741A	22	AF092810	Arg914Gln	Benign	Tolerated
A2781G	22	AF092810	Silent		
T2758C	22	AF092810	Silent		
G2907A	23	AJ419568	Silent		
A3442G	26	AY582533	Met1148Val	Benign	Tolerated
T3792C	28	AF536758	Silent		
G3817A	28	AF045016	Silent		
G3840A	28	AJ419568	Silent		

Note: The polymorphisms were identified based on sequence alignment of all *MDR1* cDNA sequences available in the GenBank/EBI/DDBJ database with the following accession numbers: AB066299, AF045016, AF092810, AF403240, AF536758, AJ419568, AY582533, DQ068953 and FJ617477. Potential effects of the non-silent polymorphisms were evaluated by SIFT and PolyPhen algorithms. Both programs consistently predicted the Gln532Arg polymorphism located in proximity to the ABC signature motif to functionally affect the P-gp transport function.

## ABBREVIATIONS

ABC: = ATP-binding cassette
CNS: = Central nervous system
GABA: = Gamma-aminobutyric acid
MDR1: = Multidrug resistance
P-gp: = P-glycoprotein
